# Wheelchair mobility performance of elite wheelchair tennis players during four field tests: Inter-trial reliability and construct validity

**DOI:** 10.1371/journal.pone.0217514

**Published:** 2019-06-06

**Authors:** Thomas Rietveld, Riemer J. K. Vegter, Rienk M. A. van der Slikke, Aldo E. Hoekstra, Lucas H. V. van der Woude, Sonja de Groot

**Affiliations:** 1 University of Groningen, University Medical Center Groningen, Center for Human Movement Sciences, Groningen, The Netherlands; 2 Human Kinetic Technology, The Hague University of Applied Sciences, The Hague, The Netherlands; 3 Royal Dutch Lawn Tennis Association, Almere, The Netherlands; 4 University Medical Center, Center for Rehabilitation, Groningen, The Netherlands; 5 Amsterdam Rehabilitation Research Center Reade, Amsterdam, The Netherlands; University of Illinois at Urbana-Champaign, UNITED STATES

## Abstract

The purpose of the current study was to assess the inter-trial reliability and construct validity (talented juniors vs. international adult players) of four wheelchair tennis field tests using inertial measurement units (IMUs). Twenty-one elite wheelchair tennis players completed four tests, which evaluate the sprinting and manoeuvrability abilities in wheelchair tennis. During all tests 3 IMUs were attached to both wheels and the frame of the athlete’s wheelchair. The IMUs enabled analysis of individual test dynamic characteristics, i.e. the linear/rotational velocity and acceleration data, as well as detected pushes. All tests showed high ICCs (0.95–0.99) for the inter-trial reliability for the IMU-based end times and also the construct validity was good, i.e. talented juniors could be discriminated from international adults. Also, velocities and accelerations during the tests could be consistently visualized, meaning that differences in test performance among participants could be designated. Within the experimental context, the field tests could be regarded as reliable and valid. With the use of IMUs it is possible to verify more detailed performance characteristics, visualize the test execution, as well as differentiate between a talented junior and international adult group and within individuals over time.

## Introduction

Wheelchair tennis became part of the Paralympic Games in 1992 and has grown in interest ever since [1]. In 2016 participants from over 100 countries participated in wheelchair tennis at varying levels and as such it is one of the fastest growing sports for people with a disability [2]. The rules in wheelchair tennis are similar to the able-bodied variant, except that with each tennis stroke an additional ball bounce is permitted. One of the main differences between wheelchair tennis and able-bodied tennis is the wheelchair, which provides an extra set of constraints onto the athlete [3].

An important aspect of wheelchair sports is the wheelchair mobility performance, defined as the wheelchair-athlete ability on court [4]. Wheelchair tennis, wheelchair basketball and wheelchair rugby are all dynamic wheelchair court sports, where the wheelchair, the wheelchair-athlete interface and the athlete’s ball skills and abilities together define the athlete’s performance [3]. The tennis racket is an important additional constraint of wheelchair tennis compared to other wheelchair sports, impacting the wheelchair mobility performance [5].

To better understand the demands of the racket in wheelchair tennis, several studies have been performed using an ergometer in lab testing [5,6,7,8]. These studies provide important fundamental insights on the difficulty of coupling/decoupling of the racket/hand to the rim, and thus on speed and power production. On the other hand, apart from lab tests, valid field tests are also very important and often preferred by trainers and coaches, since they involve play-related wheeling skills, such as sprinting, turning or stopping [9].

Although in wheelchair basketball [4,10] and rugby [11] the validity and reliability of several field tests have already been shown, it is not possible to simply use the aforementioned tests for wheelchair tennis, due to their sports-specific nature. In the wheelchair basketball tests in some parts of the test a ball needs to be thrown [4,10] and the wheelchair rugby test is specifically developed for the demands in a rugby match [11]. Combined with the use of a racket in wheelchair tennis and the amount of turns made after hitting a ball in tennis, makes it necessary to develop specific field tests for wheelchair tennis [5]. Research on reliable/valid field tests in wheelchair tennis is scarce, with only the interval shuttle wheelchair test outcomes relating to the athlete’s ranking and partly explaining the peak oxygen uptake in a standardised peak wheelchair exercise test [12]. For this reason, the Royal Dutch Lawn Tennis Association (KNLTB) has developed four field tests especially for wheelchair tennis, to evaluate the wheelchair mobility performance of their players. These are based on able-bodied tennis and wheelchair basketball tests and constitute a 20m Sprint, Spider test for manoeuvrability test, a Butterfly-sprint test and the Illinois test [10,13,14].

Inertial measurement units (IMUs) can be used to determine more detailed wheelchair mobility performance outcomes than performance times only [15]. With the use of three IMUs attached to the individual’s wheelchair, dynamic characteristics such as acceleration, angular velocity and rotational velocity, as well as push characteristics can be determined. Using this method, wheelchair mobility performance during a field test or even during a game can be described in a much more detailed way [16].

The aims of the current study are to assess the inter-trial reliability and construct validity of IMU-based wheelchair mobility performance outcomes of four wheelchair tennis field tests and assess the added value of each test. Therefore, the developed tests will first be evaluated on the reliability of the end times between the different trials of the tests. Secondly, the construct validity will be investigated with the use of a talented junior and an international adult group, in which it is expected that the international adult group will achieve higher velocities and accelerations and subsequently have faster a shorter duration. In addition, the association among the end times of the wheelchair tennis field tests will be studied to investigate the added value of each test.

## Materials and methods

### Participants

Twenty-one elite wheelchair tennis players participated in this study. Twelve players were talented youth players (juniors), selected by the Royal Dutch Lawn Tennis Association (KNLTB), the other nine adult players were playing at an international competition level (adults). The international players had an ITF ranking for men and women, while the junior group had a ranking for boys and girls. Two participants from the junior group had no ranking yet. The participants’ characteristics are summarized in Table 1. All tests and protocols were approved by the Ethical Committee of the Centre of Human Movement Sciences, University Medical Centre Groningen, University of Groningen (ECB_2017.03.17_1). All participants gave informed consent prior to participation.

**Table 1:** Participant characteristics.

Personal characteristics		N or mean (SD)	
		Adults	Juniors
Men/Women (N)		5/4	4/8
Right/Left-handed (N)		8/1	10/2
Age (years)		27.3 (8.7)	14.8 (1.5)
Body mass (kg)		68.1 (10.5)	47.6 (11.2)
Height (m)		1.75 (0.08)	1.62 (11.2)
Body Mass Index (kg/m^2^)		22.2 (3.6)	17.8 (3.1)
Impairment			
	Amputation	3	2
	Spina Bifida	1	6
	Uneven legs	2	1
	Spinal Cord Injury	2	1
	Hip dysplasia and hip displacement	1	
	Scoliosis		1
	Weak bones		1
ITF ranking*		16 (32)	12.5 (16)
Wheelchair tennis experience (years)		13.1 (7.7)	3.9 (2.0)

* Median (IQR), ITF ranking is based on men/women for adults and boys/girls for the juniors.

### Design

**Fig 1:**
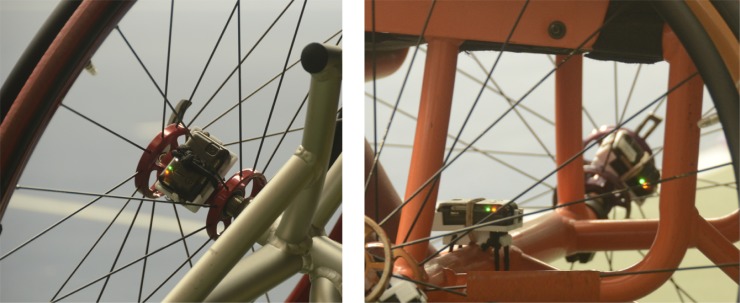
Placement IMUs, Left: Wheel, Right: Frame.

A cross-sectional design was used where all participants completed four wheelchair skill tests, which were developed for wheelchair tennis. On one occasion each of the skill tests were performed two or three times (trials). In all tests inertial measurement units (next generation IMU, Bristol, UK) were placed on both wheels and frame of the wheelchair (Fig 1), which has been shown to be a reliable way to measure wheelchair kinematics during games and wheelchair skill performance testing [15,16,17,18]. All tests were performed on indoor slow hardcourt tennis courts at two different locations, one location for each group, in the Netherlands. All participants used their own tennis wheelchair and racket. From the junior group all participants used a wheelchair with 25 or 26-inch wheels, except one player who used 24-inch wheels. From the adult group 3 participants used 26 inch, and 6 participants used 27-inch wheels. The camber angle was set at 20^o^ for all participants in both groups. Participants were tested in April, May and September 2018.

### Field tests

**Fig 2:**
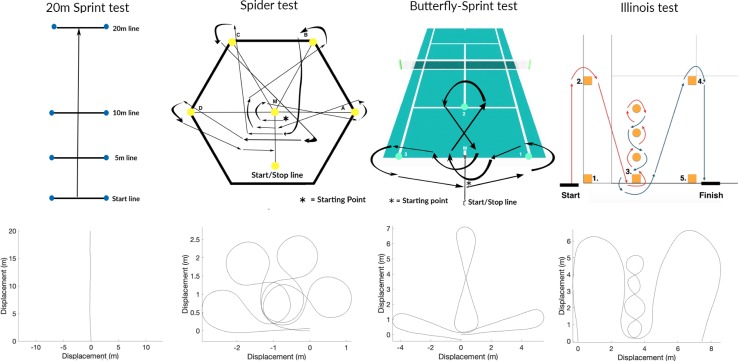
Top: Figures test, Bottom: Plots Matlab, Left: 20m Sprint test, Middle-Lleft: Spider test (Distance AM = BM = CM = DM = AB = BC = CD = 1.2m), Middle-Right: Butterfly-sprint test, Right: Illinois test (Distance pylon 1–2 = pylon 4–5 = 5.5m, pylon 1–3 = pylon 3–5 = 2.7m, Distance between the pylons for the slalom was 1.2m).

In this study four skill-oriented field tests were conducted 2 times: a 20m Sprint test [10], a Spider test for manoeuvrability [10], a Butterfly-sprint test [14], and the Illinois test [13] (Fig 2). All tests were completed while athletes held their racket. The junior group completed all tests, the adult group completed all tests, except the Butterfly-sprint test, due to time constraints. To reduce possible learning effects there was a practice session before the official test started. After each trial there was at least a resting period of 2 minutes. All trials were analysed separately, no average times per test were calculated.

### 20m Sprint test

The participant stood still with the caster wheels behind the starting line, after which a 20m over-ground straight sprint was performed, with pylons at the start, 5m, 10m and 20m point (Fig 2, left).

### Spider test (manoeuvrability)

The participant again stood still with the caster wheels behind the starting line, facing pylon D, followed by a manoeuvrability track which was completed as fast as possible (Fig 2, middle-left).

### Butterfly-sprint test

The participant stood still with the caster wheels behind the starting line facing pylon 1, after which the track was completed as fast as possible (Fig 2, middle-right).

### Illinois test

The participant again stood with the caster wheels behind the starting line and followed the arrows in the figure to complete the test (Fig 2, right).

### IMU data collection & analysis

All IMU (next generation IMU, Bristol, UK) data were resampled to a sampling frequency of 100Hz. The linear acceleration data of the frame and angular velocity data of the wheels were low-pass filtered with a recursive Butterworth filter with a cut off frequency of 10Hz [15]. Data of the junior group were collected by turning the IMUs on and off after each test. Wheel data were synchronized based on a cross-correlation of the gyroscope signals. The IMU on the frame was synchronized with the wheels based on a cross-correlation of the gyroscope of the wheels and the accelerometer of the frame. The cross-correlation is accurate at the 0.01s level, this synchronization method was chosen due to problems with the planned synchronisation, which was used with the adult players. Data of the adult group were collected by keeping the IMUs on during the whole protocol. Data were again synchronized with the cross-correlation function, although based on a previous selected part of the data at the beginning and end of the data file. The 3 IMUs were placed on top of each other after which a detectable pattern was made.

Trial times of each test were based on the IMU data, which is a reliable and accurate way [15,19]. Time started when the velocity reached a value of 0.1 m/s (Eq 2). In the 20m Sprint test the end time was based on the moment where a distance of 20 meters was covered by the wheels. Distance was obtained with the integration of angular velocity times the persons individual wheel radius. In the other three tests the end time was set based on analyses of the plots created with Matlab. A plot was made based on a combination of the gyroscope data in the Z-plane and the displacement of the wheelchair, based on the integration of the velocity signal (Fig 2, bottom). A zero-line was set at the starting point, when this line was crossed at the end of the trial, time was automatically identified.

Forward acceleration, forward velocity and travelled distance were calculated based on the algorithms developed by Van der Slikke et al. [17]. With the use of the IMU on the frame the forward acceleration (AccXFrame [m/s2]) was directly measured. Using the gyroscope data in the Y direction (GyroY [°/s]), the angular velocity was also directly measured. Due to the camber angle of the sport wheelchair and horizontal rotations of the wheelchair, the angular velocity was corrected using the sinus on the camber angle and the gyroscope data of the frame sensor in the Z-direction (GyroZFrame) [20] (Eq 1). The forward velocity was calculated using the wheel circumference (WC [m]) and the corrected angular velocity [15] (Eq 2). The travelled distance was calculated by taking the integral of the forward velocity signal.

AngVel=GyroY±sin(∝camber)*GyroZFrame (1)

$$Vel=\frac{WC}{360}*AngVel$$ (2)

The rotational velocity (RotVel [°/s]) was obtained from the gyroscope of the frame in the z-direction (GyroZFrame). The rotational acceleration (RotAcc [°/s2] was calculated by taking the derivative of the rotational velocity signal.

### Wheelchair mobility performance variables

The wheelchair mobility performance variables that were determined per trial are listed in Table 2. For each trial of all four field tests the mean and peak velocity and acceleration were calculated over the whole trajectory of the test. For the 20m Sprint test also the peak velocity and acceleration over 5, 10 and 20 meters were calculated, as well as the positions/distances where the peak velocity and acceleration were achieved.

**Table 2:** Collected wheelchair mobility performance variables.

Variables	Description
**Velocity**	V_mean_ Mean velocity on whole test [m/s]
	V_peak 5/10/20m_: Peak velocity first 5/10/20 meters [m/s]
	Pos_V peak_: Position of the peak velocity [m]
	V_peak_: Peak velocity on whole test [m/s]
**Acceleration**	a_peak 5/10/20 m_: Peak forward acceleration first 5/10/20 meters [m/s^2^]
	Pos_a peak_: Position of peak forward acceleration [m]
	a_peak_: Peak forward acceleration on whole test [m/s^2^]
**Push characteristics**	Np: Number of pushes
	D_p1,2,3_: Displacement after first, second, third push [m]
	CT_mean_: Mean Cycle Time [s]
**Rotational velocity/acceleration**	RotV_mean right/left_ Mean rotational velocity during right/left turn
	RotV_peak right/left_: Peak rotational velocity during right/left turn
	Rota_peak_: Peak rotational acceleration

For the 20m Sprint test the Velocity, Acceleration and Push Characteristics were calculated, For the Spider test and Illinois test the Velocity, Acceleration (Except V_peak5/10/20,_ a_peak5/10/20m_, Pos_V peak_, Pos_a peak_) and Rotational velocity/acceleration were calculated.

Due to the importance of rotation for the Spider, Butterfly-sprint and Illinois test, also rotation variables were calculated for these tests. The mean and peak rotational velocity were calculated for the right and left wheel separately over the whole trajectory of the test. At last also the peak rotational acceleration over the whole trajectory was calculated.

**Fig 3:**
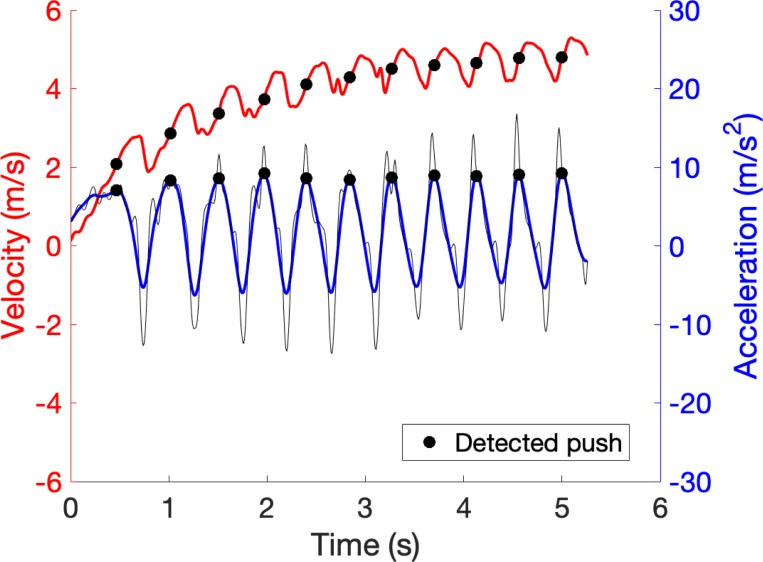
Push detection based on study of Van der Slikke et al. (2016b). The marked points represent the detected pushes. The thin/dark line is the raw acceleration signal. The blue/thicker line through the thinner line is the filtered acceleration signal. The red/rising line is the velocity signal.

Using a push detection algorithm, certain push characteristics were collected for the 20m Sprint test, since this detection algorithm is only validated for linear sprinting [19]. This push detection algorithm allowed to detect the number of pushes by automatically analysing the peaks of the acceleration signal in Matlab using the find peaks function [19]. The time between the different peaks, resulted in the cycle time (Fig 3) [19]. At last also the distance covered after the first three pushes were registered, since this gives important information about the initial acceleration [6].

### Statistical analysis

Inter-trial reliability was assessed based on the end times for each of the 4 tests by the absolute and relative reliability. To investigate the absolute reliability a paired t-test assessed the difference between the first and second trial. Bland Altman plots with 95% limits of agreements (95% LoA’s) were used as an extra control method. For the relative reliability the intraclass correlations (ICC) and standard error of the mean (SEM) were calculated. For the ICC a Two-Way Random ANOVA model, with type absolute agreement and a single measurement were used. ICC were evaluated as small (0.20), medium (0.50) or large (0.80 and higher).

Construct validity was assessed through discriminative validity, i.e. the differences between end times of the junior and adult wheelchair tennis players. Three repeated measures ANOVAs were conducted for the 20m Sprint, Spider and Illinois tests, respectively, with between subject factor ‘Playing level’ (Adults, Juniors) and within subject factor ‘trial’ (trial 1, trial 2). Partial eta-squared effect size (ES) and observed power (OP) were calculated. ES were evaluated as small (0.01), medium (0.06) and large (0.15 and higher). OP was considered large if > 0.80.

To determine the construct validity of the variables listed in Table 2, (rotational) velocity/acceleration and push characteristics, a MANOVA was used with the mean values of the wheelchair mobility performance variables as the dependent variables and the playing level as the independent variable. If the Multivariate test was significant, univariate analysis was used to analyse all variables separately. Given the linear movement, for the 20m Sprint test all variables except the rotational variables were included, since the sprint is only going forward. For the Spider and Illinois tests all variables were included except the push characteristics and the velocities for 5, 10 and 20 meters. The push characteristics were excluded since push detection is only validated for straight line sprinting [19].

The associations among the average trial times of the four different tests were evaluated with the use of Pearson correlations. A correlation was considered large when r > 0.75.

Statistical significance was set at P < 0.05 for the reliability and validity analyses, as well as the MANOVA. For the univariate analysis of the MANOVA the statistical significance was set at P < 0.004 for the 20m Sprint and P < 0.006 for the Spider and Illinois test, based on the Bonferroni correction. All data were analysed using SPSS version 22 for mac (SPSS Inc., Chicago, Illinois, USA).

## Results

The descriptive statistics of all participants are shown in Table 3. For one participant of the adult group the data of the Spider test was lost due to an empty battery of the IMU. Scores on the other tests of this participant were included in all the analyses.

**Table 3:** Reliability measures for the end times of each trial of the 20m Sprint, Spider, Butterfly-sprint and Illinois tests (Mean, SD (s)) in the elite wheelchair tennis players.

	n	Trial 1	Trial 2	Mean difference	95% LOA	ICC (CI)	SEM	t	p
**20m sprint (s)**	21	6.68 (0.94)	6.63 (0.92)	-0.05 (0.16)	-0.36 to 0.25	0.99 (0.96–0.99)	0.03	1.57	0.13
**Spider test (s)**	20	17.98 (2.14)	17.79 (2.10)	-0.19 (0.63)	-1.43 to 1.05	0.95 (0.89–0.98)	0.14	1.37	0.19
**Butterfly-sprint test (s)**	12	16.85 (1.32)	16.76 (1.30)	-0.09 (0.42)	-0.91 to 0.72	0.95 (0.84–0.99)	0.09	0.77	0.46
**Illinois test (s)**	21	21.33 (2.61)	21.04 (2.46)	-0.29 (0.49)	-1.25 to 0.68	0.98 (0.92–0.99)	0.08	2.66	0.02

The absolute reliability was assessed with a paired t-test, mean difference and 95% LOA (limits of agreement), while the relative reliability is given by the ICC (intra class correlation) and SEM (standard error of the mean).

### Reliability

**Fig 4:**
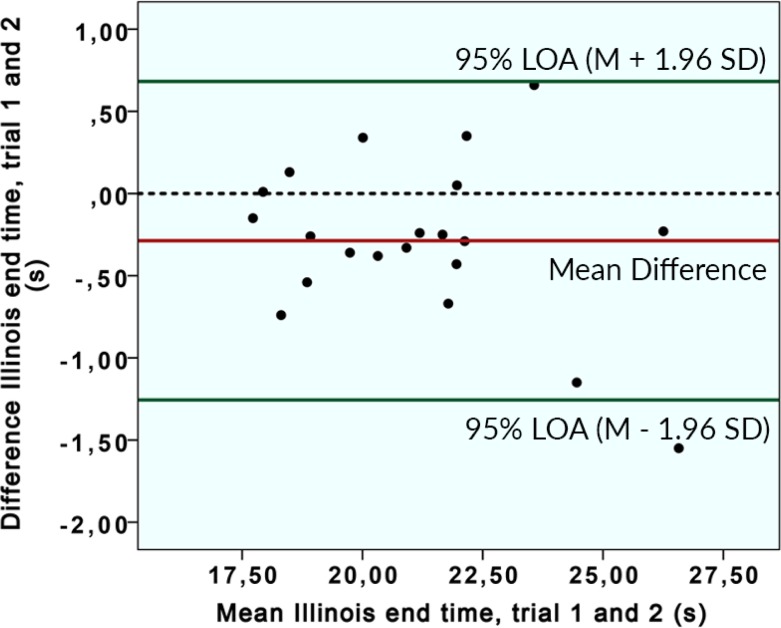
Bland Altman plot, The difference between the first and second trial against the mean of the end times (s) of the Illinois test (n = 21).

The results of the reliability analysis are shown in Table 3. The paired-samples t-test showed significant differences for the two trials of the Illinois test, in which the last measurement showed shorter durations. This could also be seen in the Bland Altman plot (Fig 4). For the 20m Sprint, Spider and Butterfly-sprint no significant differences were seen based on the paired-samples t-test. All test items scored an excellent relative reliability, high ICC (> 0.9) and small 95% limit of agreements. The SEMs were also low for all four tests.

### Construct validity of end times

End times of all three field tests differed between the junior and adult group. The adult group was faster on the 20m Sprint, Spider and Illinois tests, compared to the junior group (Table 4).

**Table 4:** Construct validity analysis of each trial of the 20m Sprint, Spider and Illinois tests (Mean, SD (s)) with the two playing levels as criterium in the group comparison.

	Adults (n = 9)		Juniors (n = 12)		Group effect			
Test	Trial 1	Trial 2	Trial 1	Trial 2	F (1,19)	p	ES^+^	OP^++^
**20 m Sprint (s)**	5.90 (0.64)	5.89 (0.56)	7.27 (0.65)	7.18 (0.73)	21.45	< 0.001*	0.53	0.99
**Spider test (s)**	16.18 (1.10)	16.21 (0.86)	19.19 (1.79)	18.84 (2.03)	15.15	< 0.01*	0.46	0.96
**Illinois test (s)**	19.18 (1.18)	18.98 (1.20)	22.94 (2.20)	22.59 (1.97)	22.82	< 0.001*	0.55	1.00

*P < 0.05

+ Effect size, partial eta squared

++ Observed Power

### Construct validity of wheelchair mobility performance variables on the 20m Sprint, Spider and Illinois tests

The descriptive statistics and results of the MANOVA for the wheelchair mobility performance variables are shown in Tables 5 and 6. A significant main effect between the two groups could be seen for the 20m Sprint, Spider and Illinois tests. The 20m Sprint test showed differences between the scores of the junior and adult group on all variables included in the analyses, except the position of the peak velocity and acceleration, the number of pushes and distances after the first three pushes. In all cases the adult group scored better compared to the junior group. The Spider test showed differences between the scores of the junior and adult group between all variables, except the peak velocity. Again, in all cases the adult group scored better. The Illinois test showed differences in scores for the junior and adult group for all variables. In this test the adult group also scored better compared to the junior group.

**Table 5:** Outcomes (Mean, SD) of the MANOVA of the wheelchair mobility performance variables for the 20m Sprint test.

	20m Sprint		F (1,19)	p	ES+	OP^++^
	Adults (n = 9)	Juniors (n = 12)				
**Main**_**effect**_			11.40	<0.01*	0.96	0.99
**V**_**mean**_	3.44 (0.33)	2.80 (0.23)	26.92	<0.001**	0.59	1
**V**_**peak5m**_	3.88 (0.38)	3.16 (0.28)	25.55	<0.001**	0.57	1
**V**_**peak10m**_	4.53 (0.47)	3.57 (0.35)	29.79	<0.001**	0.61	1
**V**_**peak20m**_	4.96 (0.54)	3.85 (0.42)	28.51	<0.001**	0.6	1
**Pos**_**V peak**_	17.82 (1.00)	17.28 (1.92)	0.46	0.51	0.02	0.10
**a**_**peak5m**_	12.33 (2.35)	6.86 (1.97)	26.51	<0.001**	0.58	1
**a**_**peak10m**_	13.13 (2.75)	6.84 (2.34)	37.13	<0.001**	0.66	1
**a**_**peak20m**_	14.23 (3.93)	7.88 (2.69)	15.49	0.001**	0.45	0.96
**Pos**_**a peak**_	11.12 (1.55)	9.09 (3.97)	0.46	0.51	0.02	0.10
**Np**	12.96 (1.42)	13.89 (1.29)	1.92	0.18	0.09	0.26
**D**_**P1**_	0.38 (0.11)	0.26 (0.06)	7.30	0.02	0.28	0.73
**D**_**p2**_	1.46 (0.26)	1.20 (0.14)	9.63	0.006	0.34	0.84
**D**_**p3**_	2.74 (0.40)	2.32 (0.26)	8.60	0.009	0.31	0.80
**CT**_**mean**_	0.42 (0.04)	0.50 (0.06)	11.11	0.003**	0.37	0.88

*P < 0.05

**P < 0.004 (Bonferroni)

+ Effect size, partial eta squared

++ Observed power

**Table 6:** Outcomes (Mean, SD) of the MANOVA of the wheelchair mobility performance variables for the Spider and Illinois tests.

	Spider test		F (1,18)	p	ES^+^	OP^++^	Illinois test		F (1,19)	p	ES^+^	OP^++^
	Adults(n = 8)	Juniors (n = 12)					Adults(n = 9)	Juniors (n = 12)				
**Main**_**effect**_			12.80	<0.001*	0.91	1.00			5.70	<0.01	0.79	0.97
**V**_**mean**_	1.74 (0.08)	1.53 (0.14)	15.21	0.001**	0.46	0.96	2.28 (0.13)	1.97 (0.16)	22.26	<0.001**	0.54	0.99
**V**_**peak**_	2.86 (0.23)	2.58 (0.34)	4.29	0.05	0.19	0.50	3.97 (0.35)	3.29 (0.36)	19.19	<0.001**	0.50	0.99
**a**_**peak**_	10.84 (2.04)	6.39 (2.28)	19.78	<0.001**	0.52	0.99	12.92 (3.61)	8.10 (3.04)	10.98	0.004**	0.37	0.88
**RotV**_**mean right**_	141 (8)	116 (12)	27.76	<0.001**	0.61	1.00	103 (7)	79 (10)	35.40	<0.001**	0.65	1.00
**RotV**_**mean left**_	130 (10)	110 (11)	18.39	<0.001**	0.51	0.98	85 (7)	74 (9)	10.01	0.005**	0.35	0.85
**RotV**_**peak right**_	257 (29)	216 (23)	12.62	0.002**	0.41	0.92	250 (25)	213 (24)	11.19	0.003**	0.37	0.89
**RotV**_**peak left**_	247 (24)	209 (19)	16.31	0.001**	0.48	0.97	253 (23)	208 (28)	15.61	0.001**	0.45	0.96
**Rota**_**peak**_	2132 (547)	1360 (232)	19.17	<0.001**	0.52	0.99	2233 (731)	1296 (218)	17.87	<0.001**	0.49	0.98

*P < 0.05

**P < 0.006 (Bonferroni)

+ Effect size, partial eta squared

++ Observed power

### Association among the end times of the field tests

The association among the end times of all four field tests were large for all tests (r > 0.75) (Table 7).

**Table 7:** Pearson correlation among end times of all four field tests.

	Illinois (n = 21)	Spider (n = 20)	Butterfly-sprint (n = 12)
**20m Sprint**	0.90	0.87	0.79
**Illinois**		0.95	0.95
**Spider**			0.97

## Discussion

In this study the inter-trial reliability and elements of construct validity of the IMU-based wheelchair mobility performance outcomes of four field tests were assessed in elite wheelchair tennis players. The reliability analysis showed good results, with a good reliability on all tests based on the ICC and SEMs, only systematic differences between the two measurement trials for the Illinois test were found. These systematic differences potentially have been caused by a learning effect of the players, thus improving between subsequent tests. As such, it would be best in future use of the Illinois test to familiarize the player with the test by doing extra practice trials or add it as part of previous training sessions, before conducting reliability analyses. The construct validity of the 20m Sprint, Spider test and Illinois test were considered good. The relation between the end times of the different field tests showed a high correlation among all four tests.

The reliability analyses indicated that the Illinois should be tested at least 2 times, due to systematic improvements. It would be best to take an average of the two trials to compensate for this systematic difference [21]. In two studies the relative reliability for the non-ball components of the tests were also considered good, which is in accordance with the results found in the current study [4,10].

The validity analyses indicated that the proposed tests could indeed be used to evaluate the wheelchair mobility performance in different levels of wheelchair tennis players. The construct validity was considered good for the 20m Sprint, Spider and Illinois tests, which means a discrimination can be made between a junior and adult player based on the end times of these tests. One study showed that high ranked players push faster, at higher speeds and cover more distance during a match compared to low ranked players, which is in accordance with the results found in our investigated field tests [22]. The found group differences can also be contributed on body mass, age and gender. A multiple regression analysis would be required with a bigger sample size to pin down personal characteristics in combination with technique and/or playing level.

According to the statistical analysis, in the 20m Sprint test the differences in end time between the junior and adult group could be contributed to higher acceleration values and hence a higher velocity, yet the position from the start at which the peak acceleration and velocity were reached was not significantly different. This indicated that probably only peak values were reached, no maximum values. The cycle time was shorter in the adult group, but the number of pushes over 20 meters were not significantly different, which indicated that the adult group were able to get more work into each push. In the Spider test the distinction between the two groups could mainly be made by the difference in rotational components, the peak velocity gave no significant differences, this is in accordance with the aim of the test to measure manoeuvrability. For the Illinois test the two groups differed significantly on all included variables, which indicated that the differences in end time between the groups were made due to a combination of all variables.

Besides using the IMUs to distinguish between players or groups, it is, of course, also very interesting to monitor a player over time regarding linear velocity/acceleration, better turning capabilities or differences in push characteristics. In the future it would be good to also assess the reliability between two measurement moments with e.g. one week apart, while also investigating the smallest detectable difference, which is important during monitoring of players [10].

**Fig 5:**
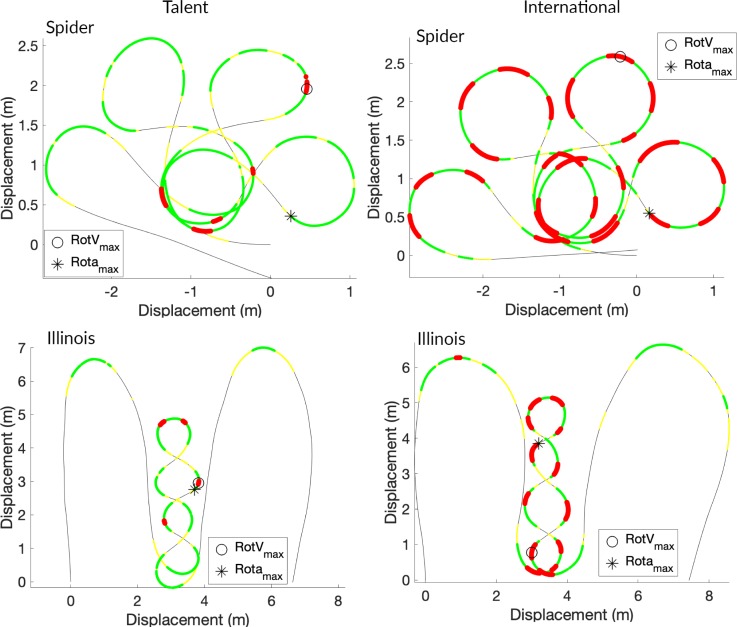
Upper-left: Spider test junior, Upper-right: Spider test adult, Lower left: Illinois test junior, Lower right: Illinois test adult. A thicker line or darker colour means a faster rotational velocity.

With the use of IMUs it has become possible to visualize a test in more detail. Where the differences between two players in a test occur, can exactly be seen. For example (Fig 5), participant 2 of the junior group and participant 8 of the adult group are compared on the Spider test and Illinois test. It can be seen that the adult player performed the Spider test with higher rotational velocities and more perfect rounds. While in the Illinois test also higher rotational velocities are visible, but the two grand turns are wider and sharper. The visualisation of the kinematic data could give trainer/coaches an easy tool to give feedback towards their players.

The wheelchair tennis field tests are easily implemented and the IMUs give detailed information about the performance of a wheelchair tennis player. In two studies the differences between wheelchair propulsion with and without a racket was shown [5,6]. This difference can also partly made visible with the use of IMUs. In the current study the rotational velocities for the right and left turn are split, but also the linear velocities could be investigated for the right and left wheel separately. With that information, studies in which a newly developed rim is evaluated [8], could also partly be tested in the field by getting an indication about the coupling/decoupling of the racket to the rim with the use of the deceleration signal. However, lab testing cannot be fully replaced, since it is the only way the velocity and resistance can be standardized.

Looking at the correlations among the end times of the 4 tests in more detail, it can be concluded that the Spider test, Butterfly-sprint and Illinois tests have such a high correlation that they are expected to measure the same components, namely the rotational components. The recommendation would therefore be to only use the 20m Sprint test, to assess the sprinting abilities, and the Spider test to measure the rotational abilities. The reason for the Spider test over the Illinois test and Butterfly-sprint test is chosen due to the duration, reliability and validity of the tests. The reliability was better, and the duration was shorter during the Spider test compared to the Illinois test, while in the Butterfly-sprint test the duration is comparable but the validity is not examined.

The current developed field tests to assess the wheelchair mobility performance of wheelchair tennis player will help to study the wheelchair characteristics (e.g. mass, tyre pressure), athlete (skills) and the interaction between both (e.g. rim). All three components determine the performance of a wheelchair athlete [3].

### Limitations and future research

Two different groups were measured on two different locations. Although both courts were hard-court, a difference in surface might have influenced the outcomes of the field tests. In the future it would therefore be interesting to see what the effect of surface on wheelchair mobility performance really is, since wheelchair tennis is played on hardcourt, grass and clay courts. Also, the tyre pressure of the junior group was not controlled at the beginning of the test. Since tyre pressure has an influence on the performance of a wheelchair athlete it is recommended to control for it in the future [23,24]. The combination of surface, tyre pressure and information about the mass of the wheelchair and user could make the comparison between a junior and adult group more correctly. The rolling resistance could be measured with the use of a coast-down test, in which it is examined how much a wheelchair decelerates [25], combined with the mass of the wheelchair and user.

Eventually the gap between lab- and field testing might be closed even more by trying to make a prediction of the power output from the IMU data, as a key indicator for performance [18,26,27]. In field testing it is difficult to standardize the power output, but for the interpretation of the outcomes of a field test power output gives essential information. It can, for example, be seen if someone is slower due to a higher power demand that needs to be overcome, due to for example body mass, or wheelchair design and maintenance.

In the current study the construct validity of the field tests is shown. Those tests were developed to test the wheelchair mobility performance of the wheelchair tennis athlete. Wheelchair tennis is more than controlling a wheelchair, also the interaction with the ball should be examined, along with the sport-specific relevance. In wheelchair basketball the use of IMUs is already investigated during match play [16]. In accordance with this study [16] it would be interesting to see what the key factors of wheelchair mobility performance in wheelchair tennis are. With that essential information it might also be possible to say if the outcomes of the field tests are comparable to a training or match situation.

## Conclusions

The wheelchair tennis field tests are reliable and valid tests to measure the wheelchair mobility performance of wheelchair tennis players. The 20m Sprint as well as the Spider test are recommended to assess the sprinting and manoeuvrability performance in wheelchair tennis athletes, based on the validity, reliability and duration of these tests. With the use of IMUs it is possible to visualize the test execution in detail and gather more detailed information to understand differences between players or monitor a player over time.

## Supporting information

S1 File Anonymous SPSS file of the data. (SAV)
